# Role of *Broad-Complex* (*Br*) and *Krüppel homolog 1* (*Kr-h1*) in the Ovary Development of *Nilaparvata lugens*

**DOI:** 10.3389/fphys.2017.01013

**Published:** 2017-12-06

**Authors:** Jianru Jiang, Yili Xu, Xinda Lin

**Affiliations:** College of Life Sciences, China Jiliang University, Hangzhou, China

**Keywords:** brown planthopper, *NlBr*, *NlKr-h1*, ovary, hormone

## Abstract

Ovarian development plays an important role in the life history of insects and is crucial for control of the insect population. The metamorphosis of an insect is precisely regulated by the interaction of the juvenile hormone and ecdysone. To understand the role of *NlBr* and *NlKr-h1* in ovary development, we used RNA interference (RNAi) to down-regulate the expression of *Broad-Complex* (*Br*) and *Krüppel homolog 1* (*Kr-h1*), two important down-stream transcription factors of juvenile hormone and ecdysone signaling. We further investigated their effects on metamorphosis and ovary development. The results showed that both *NlBr* and *NlKr-h1* are induced by ecdysone. The down-regulation of *NlBr* and *NlKr-h1* alone or together by RNAi is more effective than the topical application of ecdysone on the number of ovarioles, suggesting the necessity of *NlBr* and *NlKr-h1* in determining the number of ovarioles. The ovarian grade was significantly increased/decreased by the topical application of ecdysone and down-regulation of *NlBr* and *NlKr-h1*. The pre-oviposition period was also increased. When *NlBr* and *NlKr-h1* were down-regulated together, the ovary grade was not significantly different compared to the control (dsGFP), indicating that the development of the ovary is under the control of both *NlBr* and *NlKr-h1*. The interaction between the *NlBr* and *NlKr-h1* on the number of ovarioles and the development of the ovary indicates cross-talk between both juvenile hormone and ecdysone signaling at the transcription level in the brown planthopper. Both genes are nuclear transcription factors and may regulate signaling via down-stream genes. These results would help to both enhance the current understanding of the regulatory mechanism of the interaction between juvenile hormone and ecdysone signaling pathways during ovarian development and to design chemicals to control pests.

## Introduction

The growth and development of insects are divided into complete metamorphosis and incomplete metamorphosis. The typical characteristics of all metamorphoses are molting and metamorphosis. Juvenile hormone and ecdysone are two important hormones in the regulation of insect molting and metamorphosis (Jindra et al., 2013; Yamanaka et al., 2013; Belles and Piulachs, 2015). Juvenile hormone regulates the normal molting and metamorphosis of insects by regulating the biosynthesis and secretion of ecdysone (Riddiford et al., 2003; Riddiford, 2008). Juvenile hormone plays a role in promoting the synthesis of ecdysteroids in the larvae of insects. At the end of insect development, the concentration of juvenile hormone decreases, and ecdysone induces the expression of downstream genes, thus promoting larvae to pupae development (Yamanaka et al., 2007). Ecdysone is a class of steroid hormones mainly produced by the prothoracic glands of the larvae and adult ovaries (Thummel, 2002; Yamanaka et al., 2013; Belles and Piulachs, 2015). Ecdysone regulates downstream gene transcription through the activation of the G protein-coupled receptor signaling pathway and promotes calmodulin phosphorylation to release calcium ions into the cell, finally promoting insect metamorphosis (Cai et al., 2014). The metamorphosis of the insects is precisely regulated by the interaction of juvenile hormone with ecdysone.

Broad-Complex protein (Br) contains a Bric-a-brac/Tramtrack/Broad-Complex (BTB) domain and a zinc-finger domain. *Br* is a key transcription factor in the cross-talk between the signaling pathways of juvenile hormone and ecdysone (Zhou et al., 1998; Paul et al., 2006; Muramatsu et al., 2008; Kayukawa et al., 2016). *Br* functions in the post-embryonic development of the cockroach *Blattella germanica* and other insects (D'Avino et al., 1995; Zhou and Riddiford, 2002; Riddiford et al., 2003; Huang et al., 2013; Ureña et al., 2016).

Ovarian development, regulated by a series of hormones, plays an important role in the growth and development of insects. It is the basis for judging their physiological status and for studying the physiology of migration. Juvenile hormone is an important hormone that controls the development and maturation of the ovaries of most insects. The juvenile hormone receptors of *Pyrrhocoris apterus*, Met and Tai, are required for ovarian development. The effect of the down-regulation of the down-stream transcription factor *Krüppel homolog 1* (*Kr-h1*) on the ovarian development of insects has not been observed (Smykal et al., 2014). However, *Kr-h1* is required for the vitellogenesis and ovariole development of migratory locusts (Song et al., 2014). In *Drosophila melanogaster*, ecdysone signaling is not only required for vitellogenesis and choriogenesis during oogenesis but is also crucial for regulating the stem cell niche and primordial germ cell differentiation at the larval stage and the proliferation of germline stem cells during the adult stage (Buszczak et al., 1999; Belles and Piulachs, 2015). *Aedes aegypti Br* plays a role in the control of vitellogenesis (Chen et al., 2004).

The brown planthopper (*Nilaparvata lugens*) is a migratory agricultural pest whose migration is facilitated by an undeveloped ovary (Lin et al., 2016). Treatment of the brown planthopper with juvenile hormone greatly affects both ovarian development and the number of mature eggs (Chen et al., 2004). Ecdysone can promote the synthesis and accumulation of vitellogenin in *Bombyx mori* and promote ovarian maturation (Wu et al., 2006). The brown planthopper juvenile hormone receptor Met and its downstream transcription factor Kr-h1 are required for ovariole development and egg maturation (Lin et al., 2015).

Accumulating evidence implicates the cross-talk between juvenile hormone and ecdysone signaling in regulating insect metamorphosis at the molecular level (Zhou et al., 1998; Muramatsu et al., 2008). Juvenile hormone inhibits ecdysone secretion and its responsiveness to prothoracicotropic hormone in *Bombyx mori* (Sakurai et al., 1989), and the application of the juvenile hormone analog negatively regulates ecdysteroidogenic enzymes (Ogihara et al., 2015). In *Tribolium castaneum*, juvenile hormone inhibits the expression of *Br* gene (Zhou et al., 1998; Zhou and Riddiford, 2002), whereas ecdysone activates the expression of *Br* gene (Zhu et al., 2007).

In addition, *E75, Kr-h1, Met* and other transcription factors are also involved in the cross-talk of juvenile hormone and ecdysone signaling pathways (Zhu et al., 2007). The binding of juvenile hormone and its receptor Met activates its downstream transcription factor Kr-h1, thereby inhibiting *Br* expression to prevent metamorphosis. Juvenile hormone inhibits ecdysone-induced gene expression through *Met* in *Drosophila melanogaster* (Liu et al., 2009). However, in the cotton bollworm, the Broad protein BrZ-II is induced at high expression levels by the injection of exogenous juvenile hormone, thereby inhibiting the downstream gene expression mediated by ecdysone (Cai et al., 2014). Therefore, *Br* plays an important role in the development of insects. However, the mechanisms of the interaction between juvenile hormone and ecdysone on ovarian development remain unclear.

To understand the regulatory mechanism of the interaction between juvenile hormone and ecdysone on ovarian development, we hypothesized that the interplay between *Br* and *Kr-h1* plays an important role in the interaction between juvenile hormone and ecdysone signaling pathways on the regulation of ovarian development. We used RNA interference to down-regulate the expression of *Br* and *Kr-h1* and to investigate their effect and interaction on metamorphosis and ovary development.

## Materials and methods

### Insects

The brown planthopper population was raised on the rice seedling (*Oryza sativa* II-you-023) in the laboratory for more than 25 generations, with a culture temperature of 25°C, relative humidity of 60%, and a light cycle of 16 L: 8D. The brown planthopper was a kind gift from Professor Zhu Zengrong at the Institute of Insect Science, Zhejiang University, China.

### Total RNA extraction, cDNA synthesis and cloning

Total RNA was extracted by using RNAiso Plus (Takara, Dalian, China), according to the manufacturer's instructions. For template preparation for the qPCR analysis after dsRNA injection and expressional responses to hydroxyecdysone (ED, Cat. No. H108843, Aladdin, Shanghai, China) treatment, nymphs (20) or a mixture of long- and short-winged adults (10:10) were ground, and total RNA was extracted by phenol-chloroform extraction and isopropanol precipitation. The extracted total RNA was dissolved in DEPC-treated water, and the concentration was determined using the Nanodrop 1000 (ThermoFisher Scientific, USA). The cDNA was synthesized using the Transcriptor First Strand cDNA Synthesis kit (Roche, Shanghai, China). For spatial and temporal experiments, the eggs/nymphs/adults were collected and the brain, thorax, wing, leg, Midgut, ovary and testis of the adults were dissected as previously described (Jin et al., 2014; Lin et al., 2014). Total RNA extraction and cDNA synthesis were performed as described above.

The brown planthopper *Br* (*NlBr*) was amplified using the following primers: forward primer: CGTCATCTCGGACAGTGCTA and reverse primer: CGAAGTCCCTGAGACAA AGC. The DNA fragment was subsequently cloned into pMD18T (Takara, Dalian, China), followed by sequencing (Sangon Biotech Co., Shanghai, China).

### RNA interference and image acquisition

The 5th instar nymphs of brown planthopper were selected for silencing of the *NlBr* and *Nlkr-h1* genes. The dsRNA was synthesized with primers designed to amplify a 500-bp fragment. The primers are listed in Table 1. All dsRNAs were synthesized using the RNA Production System-T7 kit (Promega, Beijing, China). Injections were performed as previously reported (Liu et al., 2010; Li et al., 2011; Jin et al., 2014). The dsRNA injections were performed using the Narishige (MN-151) injection system. The dsRNA (0.2 μg each in a volume of 0.2 μl) was injected into the 5th instar nymph, and the same amount of *GFP* dsRNA was injected as a control group. The brown planthopper was then cultured with rice seedlings. The surviving nymphs were recorded on the second day, and the survival rate was calculated. Images of adults with eclosion defects were acquired using Nikon microscopy (SMZ745T) and processed by Adobe Photoshop CS4. The numbers of adults with eclosion defects were recorded and calculated.

**Table 1:** Primers for dsRNA synthesis.

**Name**	**Sequence**
dsNlBrF	TAATACGACTCACTATAGGGAGACCACCGTCATCTCGGACAGTGCTA
dsNlBrR	TAATACGACTCACTATAGGGAGACCACCGAAGTCCCTGAGACAAAGC
dsNlKrhT7F	TAATACGACTCACTATAGGGAGACCACGTGGGGTTCAGTCCTGAGGA
dsNlKrhT7R	TAATACGACTCACTATAGGGAGACCACCAGTCGAACACACACCGGAG
dsGFPT7F	GGATCCTAATACGACTCACTATAGGAAGGGCGAGGAGCTGTTCACCG
dsGFPT7R	GGATCCTAATACGACTCACTATAGGCAGCAGGACCATGTGATCGCGC

To further determine the level of transcription after dsRNA injection, the expression levels of *NlBr* and *NlKr-h1* were detected by quantitative real-time PCR. After 1, 3, 5, and 7 days, brown planthoppers injected with the dsRNAs of *GFP, NlBr, NlKr-h1, NlBr* + *Nlkr-h1* were collected, and whole nymphs or adults were ground. A fragment of approximately 120 bp was designed for the quantitative detection of the expression levels of *NlMet* and *NlKr-h1*. For qPCR, three replicates were used. The reference gene was selected as previously reported (Jin et al., 2014; Yuan et al., 2014). The qRT-PCR primers used in the present study are listed in Table 2. Three replicates were used for qRT-PCR. Each reaction included SYBR Premix ExTaq (2x) 10 μL, forward primer (10 μmol/L) 0.4 μL, reverse primer (10 μM) 0.4 μL, cDNA 2 μL, and dH2O 7.2 μL, using two-step qPCR amplification programme. The data were analyzed using the 2^−ΔΔCt^ method (Livak and Schmittgen, 2001).

**Table 2:** Primers for qRT-PCR.

**Name**	**Sequence**
NlKrhQF	TGATGAGGCACACGATGACT
NlKrhQR	ATGGAAGGCCACATCAAGAG
NlBrQF	CCAGGCAAACAACCCAATC
NlBrQR	CTACACTGCCCCTCTTCACG
NlRPS15QF	TAAAAATGGCAGACGAAGAGCCCAA
NlRPS15QR	TTCCACGGTTGAAACGTCTGCG

### Ovary grading

To study ovary grading, the dsRNAs of *GFP, NlBr, Nlkr-h1, NlBr* + *Nlkr-h1* were injected, and then the newly eclosed females were cultured separately and mated with long-winged males. Two days later, the ovaries of the females were dissected in 1 × PBS buffer, and subsequently graded. The grading criteria were referenced as previously described (Lin et al., 2015). For the egg laying experiment, newly eclosed females, treated as described above, were placed in a tube containing only one rice seedling and mated with long-winged males. Starting from the next day, the numbers of eggs laid were recorded daily. Three biological repetitions were made for each experiment.

### Hormone treatment

A total of 200 brown planthoppers at the 5th instar nymph stage were selected and treated with hydroxyecdysone (ED, Cat. No. H108843, Aladdin, Shanghai, China) at a concentration of 0.05 μg/μl, and a 0.2-μl volume was administered to each nymph; acetone, administered at the same amount, was used as the control. After 1 h of treatment, the surviving nymphs were cultured on rice seedlings, and then the newly eclosed long-winged and short-winged females were collected and mated with the long-winged males. After 2 days, the ovaries of the females were dissected in 1 × PBS buffer and graded as previously described (Lin et al., 2015). At 12, 24, and 48 h after injection, the expression levels of the genes described above were measured by qRT-PCR; the experiments were performed in triplicate.

## Results

### Induction of *NlBr* and *NlKr-h1* by hydroxyecdysone (ED)

**Figure 1:**
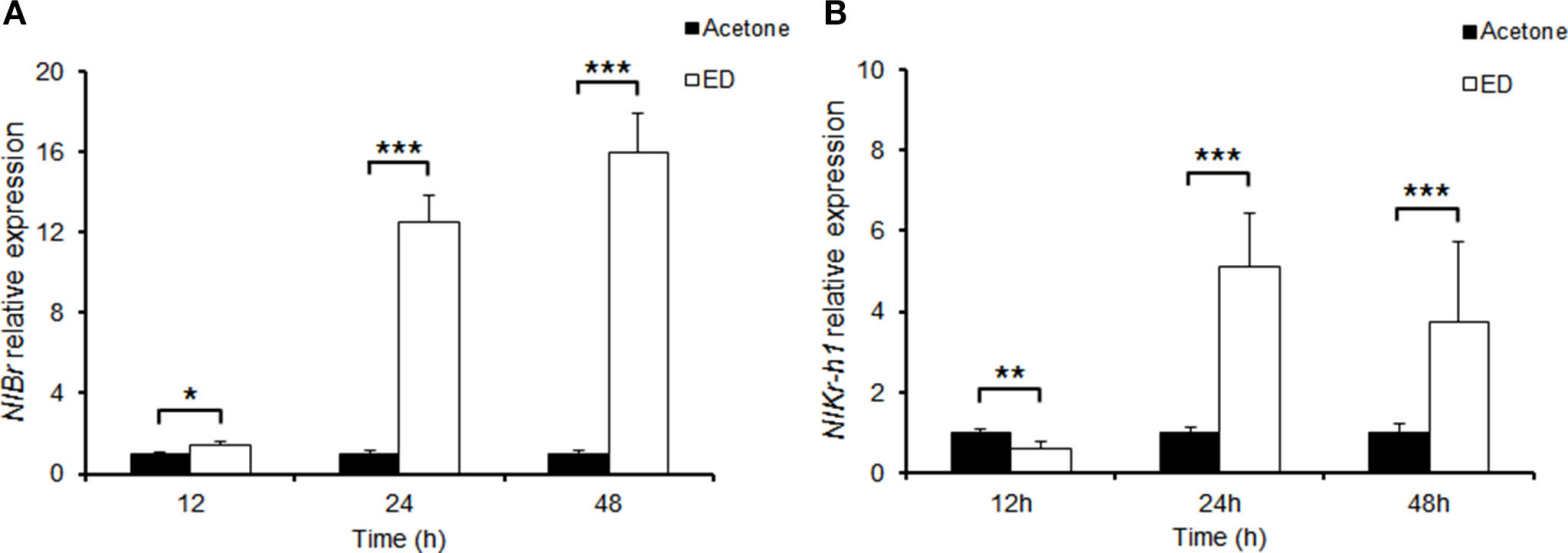
Induction of *NlBr* and *NlKr-h1* by hydroxyecdysone. **(A)** The expression of *NlBr* was induced by topical application of hydroxyecdysone (ED) after 12 (^*^P < 0.01), 24, and 48 h (^***^P < 0.001); **(B)** The expression of *NlKr-h1* was inhibited at 2 h after the topical application of hydroxyecdysone (ED) (^**^P < 0.01) and then significantly induced after 24 and 48 h (^***^P < 0.001).

We cloned brown planthopper *NlBr* into pMD18T and analyzed the coding sequence. The predicted amino acid sequence showed that the brown planthopper NlBr is conserved across species (Figure S1). This gene is particularly highly conserved in the N-terminus (Figure S2). The BTB domain at the N-terminus is highly conserved, and the Zinc-finger domain at the C-terminus is less conserved, although we observed the conserved amino acids cysteine and histidine (Figure S2). Previously, we reported that the transcription factor *NlKr-h1* downstream of the juvenile hormone signaling is induced by the treatment of juvenile hormone or its analogs (Jin et al., 2014). To test whether the downstream factors of juvenile hormone and ecdysone signaling, *NlKr-h1* and *NlBr* are induced by the ecdysteroid treatment, we compared the expression levels of *NlBr* and *NlKr-h1* in brown planthoppers treated by ecdysteroid with the control treated by acetone. The results show that *NlBr* is significantly induced by ecdysone treatment after 12, 24, and 48 h (Figure 1A). However, the expression of *NlKr-h1* is reduced by ecdysone treatment after 12 h and significantly induced by treatment after 24 and 48 h (*P* < 0.001, Figure 1B).

### The spatial and temporal expression of *NlBr*

**Figure 2:**
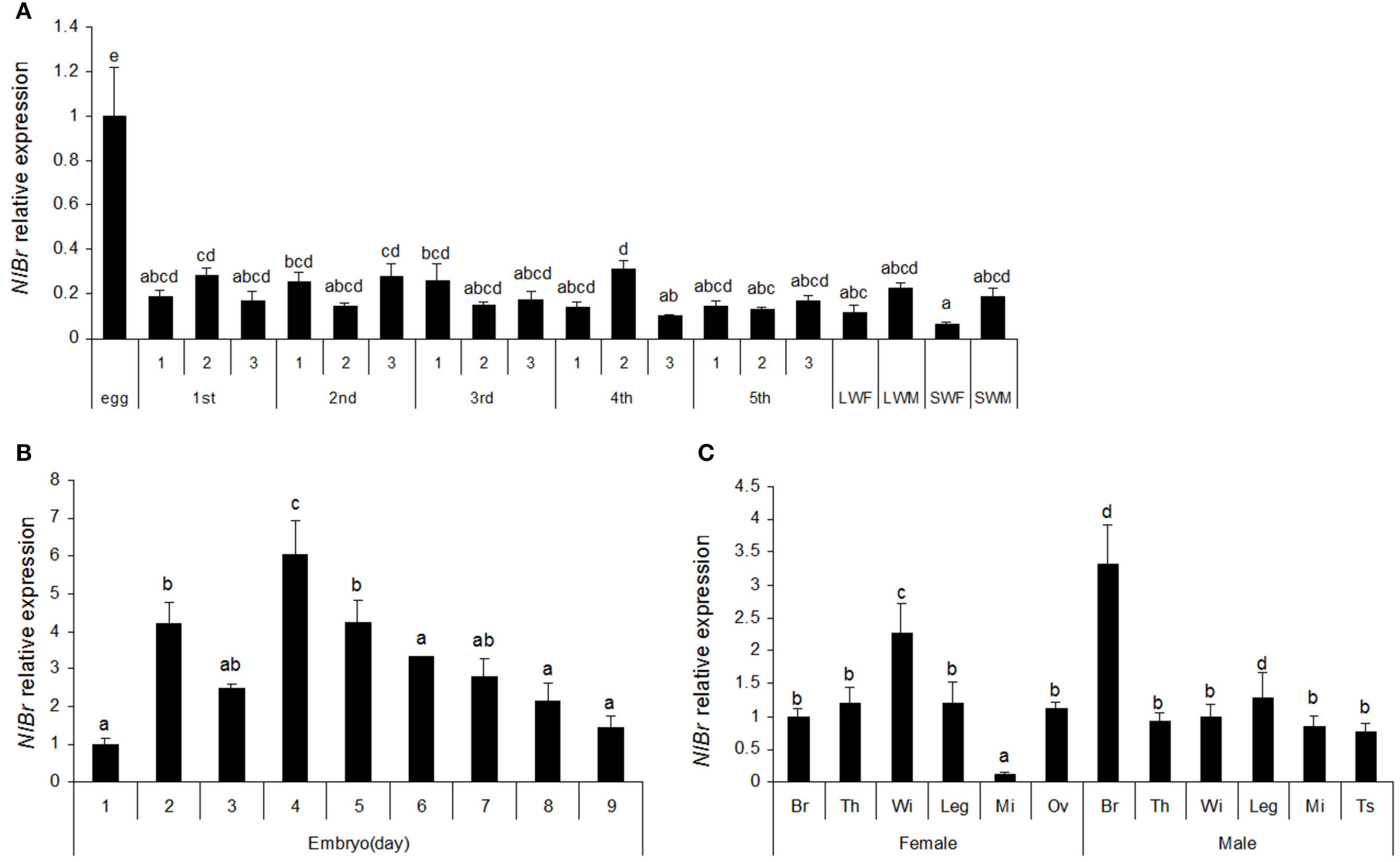
The temporal and developmental expression profiles of *NlBr*. **(A,B)** Relative expression of *NlBr* in eggs, nymphs and adults; **(C)** Relative expression of *NlBr* in various tissues. Br, brain; Th, thorax; Wi, wing; Leg, leg; Mi, Midgut; Ov, the ovary; Ts, testis. The letters indicate significant differences according to Duncan's multiple range tests at a 0.05 level.

To understand the expression characteristics of *NlBr*, we detected the expression of this gene at different developmental stages by qRT-PCR. The results showed that there were different expression levels in all stages of development (egg, nymph, and adult). The expression level was the highest on day 4 and almost identical on days 2 and 5 during egg development (Figure 2B), which was 4.2 times that of the expression observed on day 1 and relatively higher expression was detected on days 6 (3.34-fold) and 7 (2.8-fold). In the entire development process of brown planthopper, the expression of *Br* during the embryo stage was the highest (Figure 2A), which was 3 times that observed in the nymph and adult. The differences in all nymph stages were not significant, except the 4th instar nymph (Figure 2A). In both long- or short-winged adults, there was a significant difference between females and males (Figure 2A).

The expression of *NlBr* in different tissues of brown planthopper are shown in Figure 2C. In males, the expression of *NlBr* was the lowest the midgut (12.47%), while in females, the expression of *NlBr* was highest in the wing (228%). In males, the expression of *NlBr* was the highest in the brain (330%).

### Effects of down-regulation of *NlBr* and *NlKr-h1* on metamorphosis of the brown planthopper

**Figure 3:**
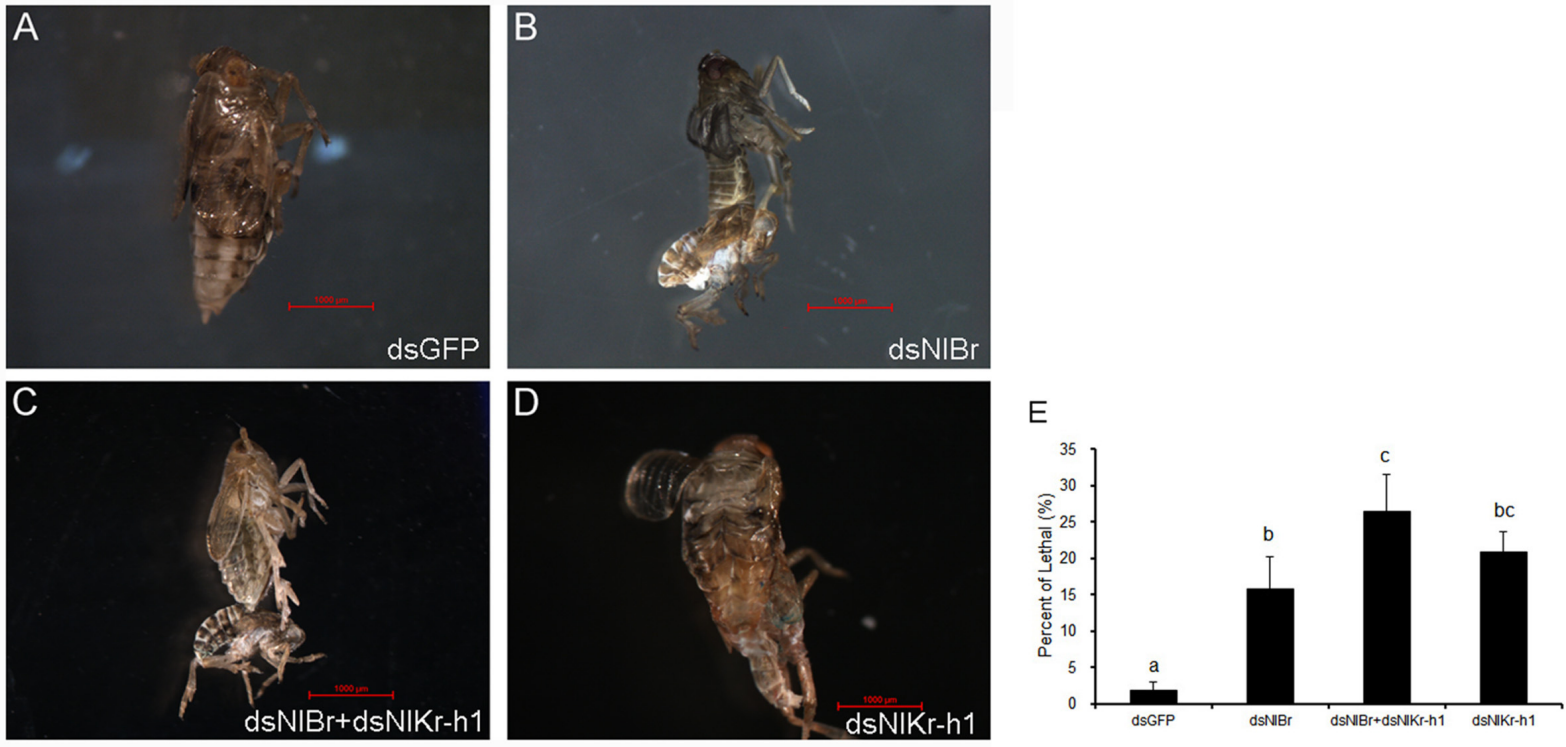
Down-regulation of *Br*/*Kr-h1* induced an eclosion defect and increased lethality during metamorphosis. **(A–D)** Representative images after injected with dsRNA. **(A)** dsGFP (control, *n* = 255); **(B)** dsNl*Br* (*n* = 79); **(C)** dsNl*Br*+dsNlKr-h1 (*n* = 213); **(D)** dsNlKr-h1 (*n* = 242); **(E)** Percentage lethality after the down-regulation of *Br* /*Kr-h1*. Duncan's multiple range test was used, different letters indicate *P* < 0.05.

When we down-regulated *NlBr* and *NlKr-h1* alone or together by RNAi, we observed defects on metamorphosis and increased eclosion lethality when compared with the control injected with dsGFP (Figure 3). Metamorphosis was disrupted at the late stages, leading to the failure of metamorphosis before eclosion (Figures 3B–D). The ratios of eclosion lethal after the down-regulation of *NlBr* and *NlKr-h1* alone or both were not significantly different (*P* = 2.05). There is no significant difference compared to the control when *NlBr* and *NlKr-h1* are down-regulated together (*P* = 2.05).

### Effects of down-regulation of *NlBr* and *NlKr-h1* on ovary development

**Figure 4:**
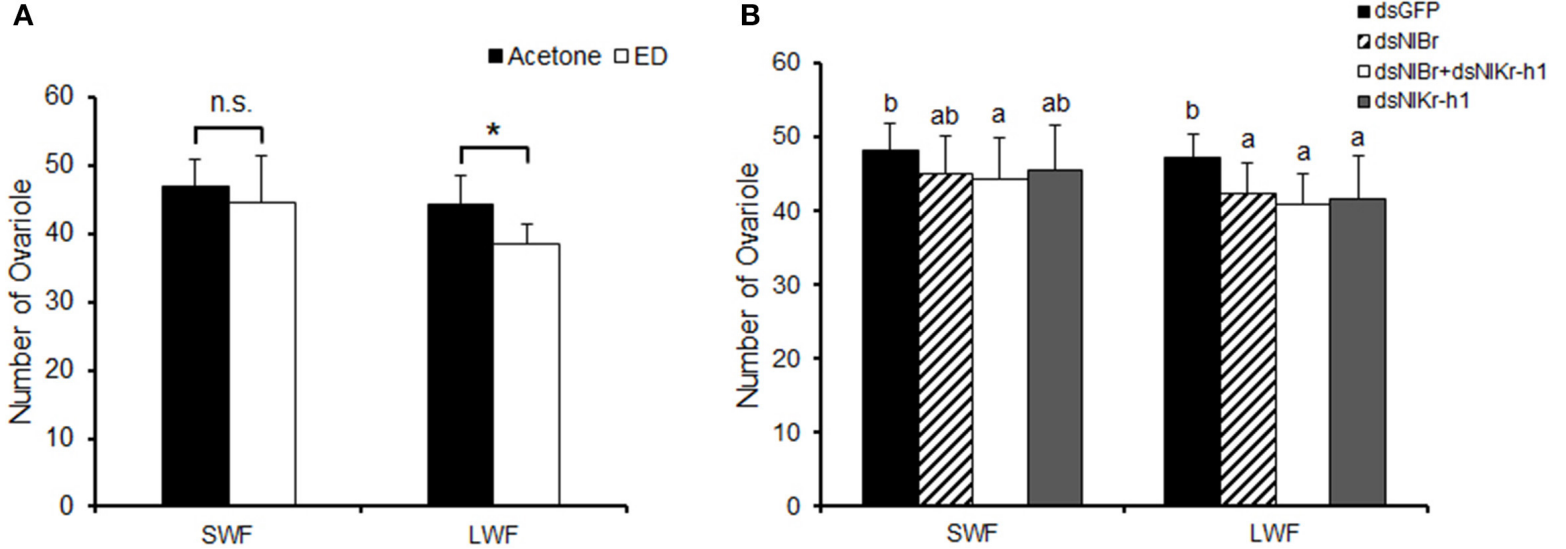
Effects of hydroxyecdysone (ED) and down-regulation of *NlBr* and *NlKr-h1* on the number of ovarioles. Hydroxyecdysone (ED) was added by topical application, and the genes were down-regulated by RNAi. SWF, short-winged female; LWF, long-winged female. **(A)** Number of ovarioles after the topical application of ecdysone, Student's *t*-test was used, ^*^P < 0.05; **(B)** Number of ovarioles after down-regulation of *NlBr*/*NlKr-h1*. The letters a–b indicates significant differences according to Duncan's multiple range tests at a 0.05 level. Number of female adults used: Hydroxyecdysone (0.05 μg/μl *n* = 21, 0.1 μg/μl *n* = 28 short-winged, 14 long-winged), Acetone (*n* = 36 short-winged, 16 long-winged), dsGFP (*n* = 40 short-winged, 40 long-winged), dsNl*Br* (*n* = 42 short-winged, 40 long-winged), dual knockdown of *NlBr* and *NlKr-h1* (*n* = 36 short-winged, 40 long-winged), and dsNlKr-h1 (*n* = 38 short-winged, 38 long-winged).

After the nymphs of brown planthopper were injected with dsRNA, the ovaries of brown planthopper were dissected and the ovarian tubules were counted. The 5th instar nymphs of brown planthopper were treated with hydroxyecdysone (0.05 μg/μl) and compared with the control group for ovary development and the number of ovarian tubules dissected after eclosion (Figure 4). There was no difference in the number of ovarian tubes after hydroxyecdysone treatment compared to the control treated with acetone in the short-winged female (SWF, Figure 4A), however these parameters were significantly different in the long-winged female (LWF, Figure 4A). The number of ovarian tubes of *NlBr* + *NlKr-h1* down-regulated females was significantly different from that of the control dsGFP in both short- and long-winged females (Figure 4). Moreover, the effects of the down-regulation of *NlBr* and *NlKr-h1* alone or together were significantly different from those of the control dsGFP in the long-winged females, but not in the short-winged form (Figure 4B). However, we observed no differences among *NlBr, NlKr-h1*, and *NlBr* + *NlKr-h1* (Figure 4).

**Figure 5:**
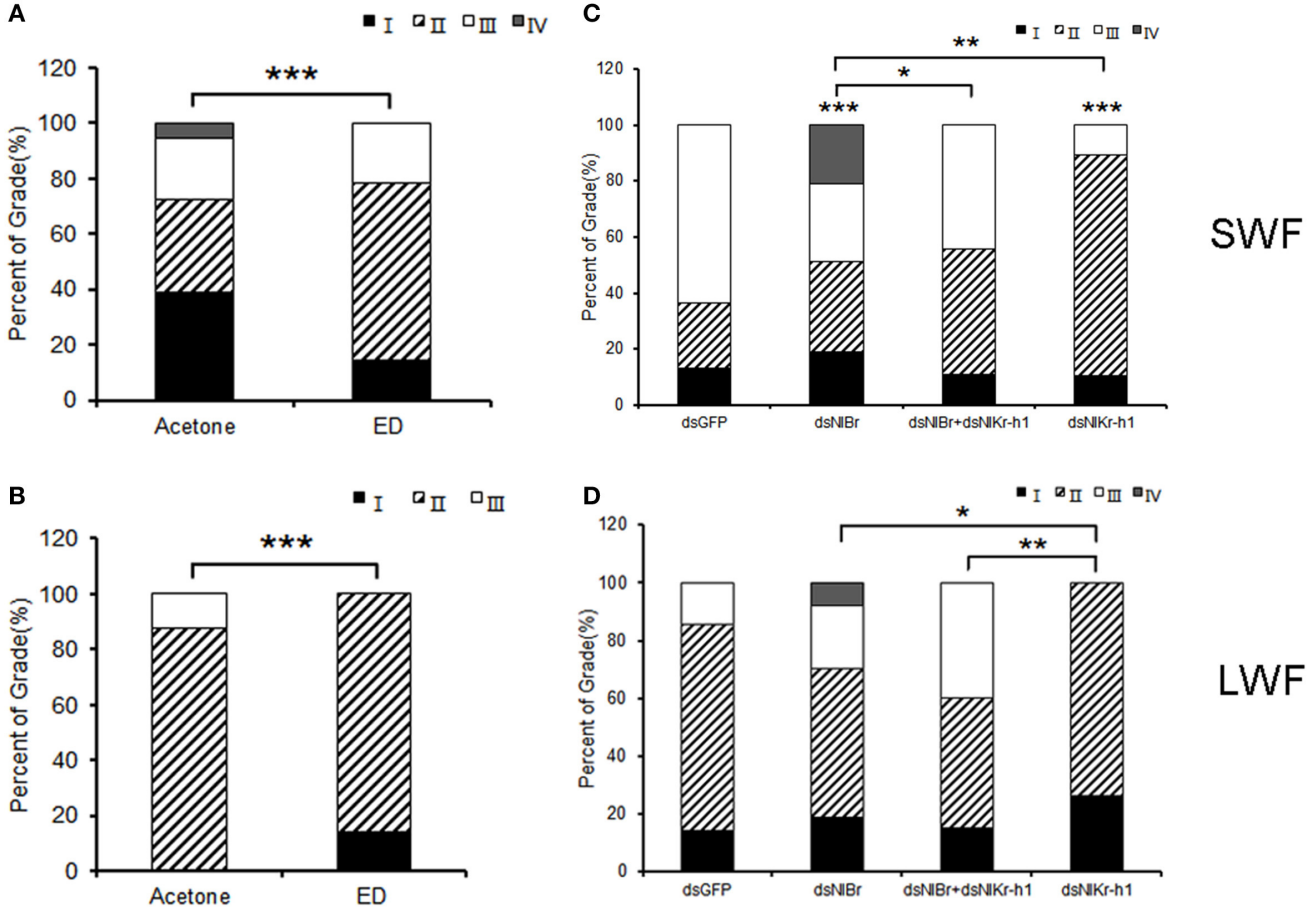
Comparison of the ovary development between the long- and short-winged females after dsRNA treatment. **(A,C)** Short-winged; **(B,D)** long-winged. I-IV, four grades of the brown planthopper ovary (Lin et al., 2015). Percentages of stages I, II, and III of ovarian development 2 days post-adult eclosion (PAE) are shown. For Chi-square test, ^*^P < 0.05, ^**^P < 0.01, ^***^P < 0.001. SWF, short-winged female; LWF, long-winged female. ^*^ Above the bar indicates the comparison between the dsRNA of *NlBr* and/or *NlKr-h1* and dsGFP, and ^*^ indicates the two treatments linked by the square brackets. Number of female adults used: Hydroxyecdysone (0.05 μg/μl *n* = 21, 0.1 μg/μl *n* = 28 short-winged, 14 long-winged), Acetone (*n* = 36 short-winged, 16 long-winged), dsGFP (*n* = 40 short-winged, 40 long-winged), dsNl*Br* (*n* = 42 short-winged, 40 long-winged), dual knockdown of *NlBr* and *NlKr-h1* (*n* = 36 short-winged, 40 long-winged), and dsNlKr-h1 (*n* = 38 short-winged, 38 long-winged).

The effect of *NlBr* and *NlKr-h1* on the development of the ovary was studied. The ovaries of females were treated with hydroxyecdysone or injected with dsNlBr, dsNlKr-h1, or dsNl*Br* + dsNlKr-h1. The grading criteria were determined as reported previously (Lin et al., 2015). Treatment of hydroxyecdysone leads to a significant delay of ovarian development in both the long-winged and short-winged female (*P* < 0.001, Figures 5A,B). The down-regulation of either *NlBr* or *NlKr-h1* significantly affected ovarian development (Figures 5C,D). In the short-winged female, the down-regulation of *NlBr* increased ovarian development, whereas the down-regulation of *NlKr-h1* significantly delayed the ovarian development (Figure 5C). In the long-winged female, the down-regulation of either *NlBr* or *NlKr-h1* did not significantly change ovarian development, despite the significant difference between these genes. When both genes were down-regulated, the development of the ovary revealed no significant difference from that of the control injected with dsGFP. Moreover, in both short- and long-winged females, the ovarian development of *NlBr* and *NlKr-h1* down-regulated adults was significantly different, indicating the different effects of the juvenile hormone and ecdysone signaling in regulating the development of the ovary in the brown planthopper.

### Effects of down-regulation of *NlBr* and *NlKr-h1* on egg number and pre-oviposition period

We next examined whether the number of eggs and pre-oviposition period of females are changed after the emergence of 5th instar nymphs by the down-regulation of *NlBr, NlKr-h1*, or *NlBr* + *NlKr-h1*.

**Figure 6:**
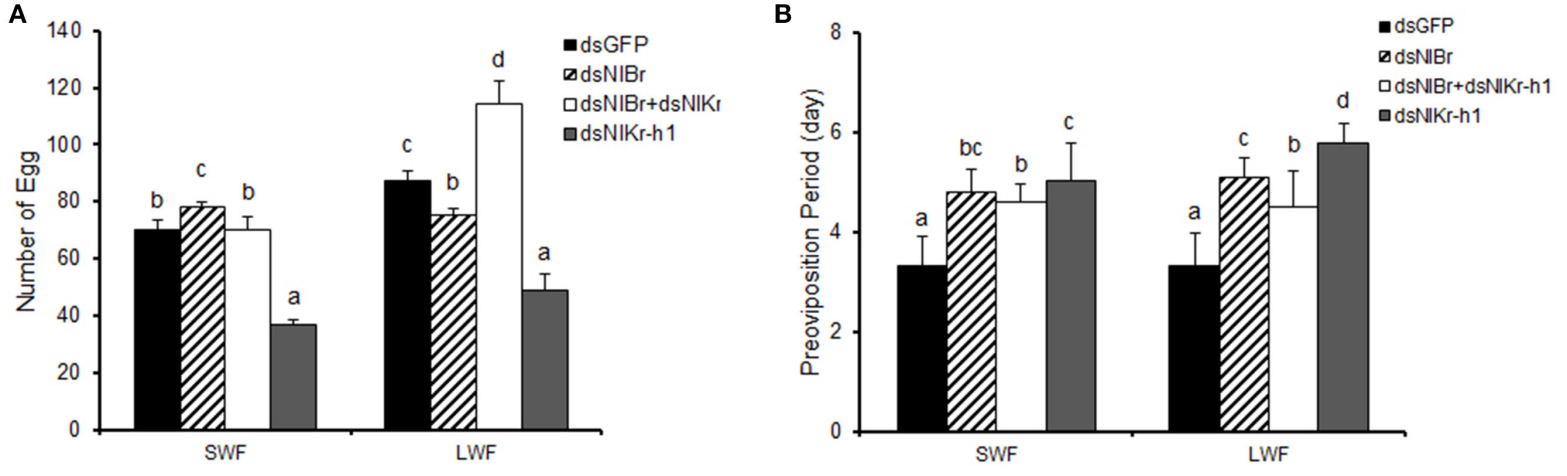
Comparison of the egg numbers and pre-oviposition periods between the long- and short-winged females after dsRNA injection. **(A)** number of egg; **(B)** preoviposition period (day). SWF, short-winged female; LWF, long-winged female. The letters a–d indicate significant differences in either SWF or LWF after the injection of dsRNAs according to Duncan's multiple range tests at the 0.05 level. Number of females used: dsGFP (*n* = 21 brachypterous and 17 macropterous), dsNl*Br* (*n* = 27 brachypterous and 28 macropterous), dual knockdown of *NlBr* and *NlKr-h1* (*n* = 18 brachypterous and 7 macropterous), and dsNlKr-h1 (*n* = 23 brachypterous and 14 macropterous).

Eggs laid after the down-regulation of *NlBr* and *NlKr-h1* alone or together at 5th instar nymphs showed significant differences compared to the control (dsGFP) (Figure 6A). The number of eggs decreased after the down-regulation of *Nlkr-h1* in both short- and long-winged females. In the females with down-regulation of *NlBr*, the number of eggs laid was reduced in the long-winged females and increased in the short-winged female. We also recorded the eggs laid by females with the down-regulation of both genes. We found a significant increase of the number of eggs in the long-winged and no significant change in the short-winged female. The pre-oviposition period was significantly increased in all females injected with dsRNAs (Figure 6B).

**Figure 7:**
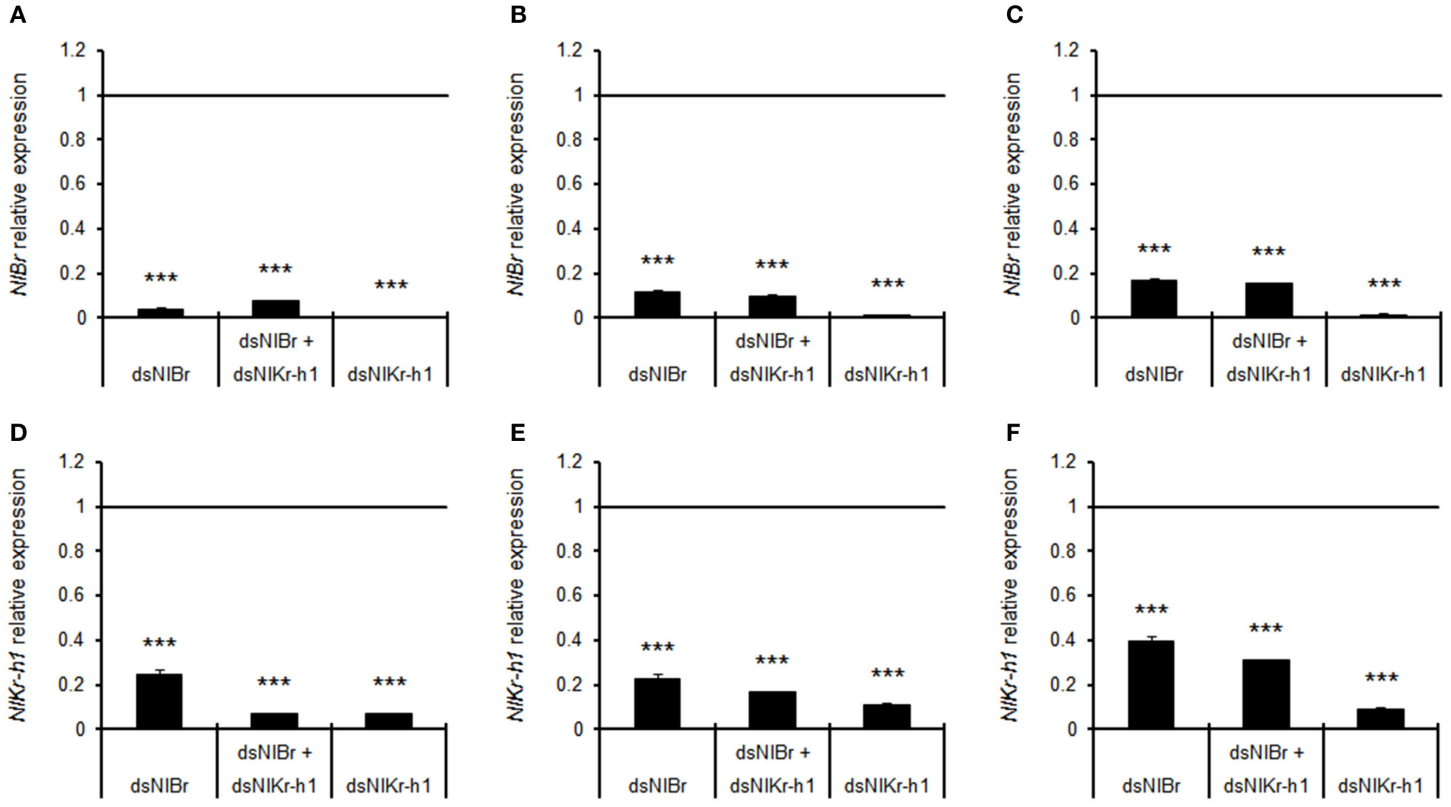
Relative expression levels of *Br, Kr-h1, Br* + *Kr-h1* after dsRNA injection in 5th instar nymphs compared to the control injected with GFP dsRNA. The expression levels were measured after the injection of dsRNA at 1 **(A,D)**, 3 **(B,E)** and 5 days **(C,F)**. Student's *t*-test was used, ^***^P < 0.001.

Quantitative real-time PCR showed that the expression levels of *NlBr* and *NlKr-h1* after the injection of *NlBr, NlBr* + *NlKr-h1*, and *NlKr-h1* dsRNAs were significantly decreased after RNA interference (Figure 7).

## Discussion

The relatively high expression of *NlBr* at the embryonic stage and the expression in almost all developmental stages indicated the requirement of this gene during embryo development and throughout the entire development period. Topical application indeed significantly increased the transcriptional level of *NlBr* and *NlKr-h1* after 24 and 48 h of treatment (Figure 1); however, the reverse responses were observed after 12 h (Figure 1). The differing responses of the expression changes of *NlBr* and *NlKr-h1* to hydroxyecdysone treatment suggest that the interplay between *NlBr* and *NlKr-h1* is more complicated. The effects of the topical application of hydroxyecdysone on the number of ovarioles were different in long- and short-winged females (Figure 4).

Further functional studies of *NlBr* on the growth and development of the brown planthopper (*N. lugens*) were performed by RNA interference. When comparing the number of ovarioles, both the effect of the topical application of hydroxyecdysone and the down-regulation of *NlBr* and *NlKr-h1* alone or together by RNAi was more effective on the reduction of the number of ovarioles in long-winged females (Figure 4B), i.e., the number of ovarioles was is not significantly changed in the short-winged female, but was significantly decreased in the long-winged female (Figure 4), except for a significant decrease in the ovariole number in short-winged female when *NlBr* and *NlKr-h1* were down-regulated together (Figure 4B). This finding indicates the necessity of *NlBr* and *NlKr-h1* for determining the number of ovarioles at least in the long-winged female. Taken together, these results suggest that when evaluating the number of ovarioles, the long-winged female is more sensitive to the topical application of the ecdysone or the down-regulation of *NlBr* and *NlKr-h1*, likely because the short-winged female is the reproductive form and is more insensitive to the topical application of the ecdysone or down-regulation of the *NlBr* and *NlKr-h1*. Additionally, nymphs were used when measuring the expression levels of *NlBr* and *NlKr-h1* after hydroxyecdysone treatment, suggesting that the future of short- and long-winged females at the 5th nymph stage could not be discriminated. Further experiments showed that the ovarian grade is more sensitive to endocrinal changes and is significantly affected by the topical application of hydroxyecdysone in both the long- and short-winged brown planthoppers. The ovary grade was significantly changed by the down-regulation of *NlBr* and *NlKr-h1* alone, especially in the short-winged adults (Figure 5C). Again, this result indicated the requirement of ecdysone, down-stream transcription factors of JH and ecdysone signaling for ovary development. The ovary grade of short-winged brown planthoppers after the down-regulation of both *NlBr* and *NlKr-h1* was not significantly changed (Figure 5C), suggesting the possible interplay of JH and ecdysone signaling via *NlBr* and *NlKr-h1*, and the potential counter-active interaction of these two transcription factors. Taken together, the differential responses of the long- and short-winged brown planthoppers are reminiscent of the variance in ovary development between the long- and short winged female adults (Kisimoto, 1965; Zera and Denno, 1997; Ayoade et al., 1999). This variation could be a result of differential mechanisms of regulating ovary development in the long- and short-winged female adult, indicating the possible existence of different interaction modes between *NlBr* and *NlKr-h1*.

Ovarian development and metamorphosis are complicated biological processes; the regulation of ovary development by juvenile hormone and ecdysone signaling could be different from that of regulating metamorphosis. In addition, although the genetic interaction among *E93, Kr-h1*, and *Br* in *Tribolium castaneum* has been reported (Ureña et al., 2016), whether there is a physical interaction between NlBr and NlKr-h1 needs further investigation.

The interaction between the *NlBr* and *NlKr-h1* on the number of ovarioles and the development of the ovary indicates cross-talk between the juvenile hormone signaling and ecdysone signaling at the transcription level. This mechanism is reminiscent of the fact that both genes are nuclear transcription factors and may regulate both signaling pathways via the regulation of down-stream genes. JH could prevent the expression of Br-C and pupae formation in *D. melanogaster* (Dubrovsky, 2005; Riddiford, 2008). In *Helicoverpa armigera*, the cross-talk between the two signaling pathways was partially mediated by phosphorylation variation of Calponi (Liu et al., 2011). Recent analysis of cis and trans elements involved in the repression of *BR* revealed that Kr-h1 inhibits insect metamorphosis via direct transcriptional repression of *Br* (Kayukawa et al., 2016). Through the down-regulation of gene expression by RNAi, we showed that the possible interaction of *NlBr* and *NlKr-h1*, i.e., reduced transcriptional expression of *Kr-h1*, leads to reduced effects on the metamorphosis of Br by a reduction of the repressible effect on the expression of *NlBr*. Although further biochemical tests are required to confirm their interaction, the present results on ovary grading support an opposite role for *NlBr* and *NlKr-h1* during the development. In addition, transcription factors control the production rate of mRNA. The *Bombyx mori Br* is a crucial factor regulating the transcription of vitellogenin (Yang et al., 2014), which is a key gene required for egg formation, indicating a potential indirect mechanism by regulating down-stream targeting genes. Therefore, deciphering the down-stream targeting genes controlled by *Br* and *Kr-h1* would enhance the current understanding of the mechanism of ovary development and egg formation in brown planthopper.

These results would help decipher the regulatory mechanism of the interaction between juvenile hormone and ecdysone on ovarian development. This information could lead to the use of the RNAi-based release of insects carrying a dominant lethal (RIDL) system strategy (Lin and Wang, 2015), or more directly, the design of chemicals to disrupt the components of JH and ecdysone signaling pathways to control this pest.

## Author contributions

XL designed the study, analyzed the data, and wrote the manuscript. JJ did the experimental work and analyzed the data. YX did the experimental work and collected the insects.

### Conflict of interest statement

The authors declare that the research was conducted in the absence of any commercial or financial relationships that could be construed as a potential conflict of interest.

## Supplementary material

Figure S1 Phylogenic analysis of Br proteins. Ln, *Lasius niger* (KMR04998.1); Em, *Eufriesea Mexicana* (XP_017755324.1); Ac, *Apis cerana* (XP_016913178.1); Si, *Solenopsis invicta* (XP_011169636.1); Mp, *Monomorium pharaonic* (XP_012538438.1); Da, *Diachasma alloeum* (XP_015108610.1); Ad, *Apis dorsata* (XP_006623320.1); Pa, *Pyrrhocoris apterus* (AEW22982.1); Ha, *Helicoverpa armigera* (AGW25457.1); Ds, *Drosophila simulans* (KMZ07685.1); Dp, *Diploptera punctate* (AIM47237.1); Cq, *Culex quinquefasciatus* (EDS39011.1); Am, *Apis mellifera* (NP_001035356.1); Aa, *Aedes aegypti* (AAS80329.1); Cc, *Ceratina calcarata* (XM_018020932); Bm, *Bombyx mori* (NP_001104804.1); Tc, *Tribolium castaneum* (BAJ16548.1); Dm, *Drosophila melanogaster* (ACZ95175.2); Ms, *Manduca sexta* (AAC78288.1); Nl, *Nilaparvata lugens* (KU640204).

Figure S2 Alignment of the amino acid sequences of *N. lugens* Br with those of its homologs in *Bombyx mori, Drosophila melanogaster, Tribolium castaneum, Manduca sexta* and *Aedes aegypti*. Domains-BTB (Red), C2H2 Zn finger (Blue) are boxed. *Bm, Bombyx mori* (NP_001104804.1); *Dm, Drosophila melanogaster* (ACZ95175.2); *Tc*, T*ribolium castaneum* (BAJ16548.1); *Ms, Manduca sexta* (AAC78288.1); *Aa, Aedes aegypti* (AAS80329.1).
