# Supplementary figures and images for: Role of Broad-Complex (Br) and Krüppel homolog 1 (Kr-h1) in the Ovary Development of Nilaparvata lugens
Source: Front Physiol. 2017 Dec 6;8:1013. doi: 10.3389/fphys.2017.01013 (PMC5724046; doi:10.3389/fphys.2017.01013)

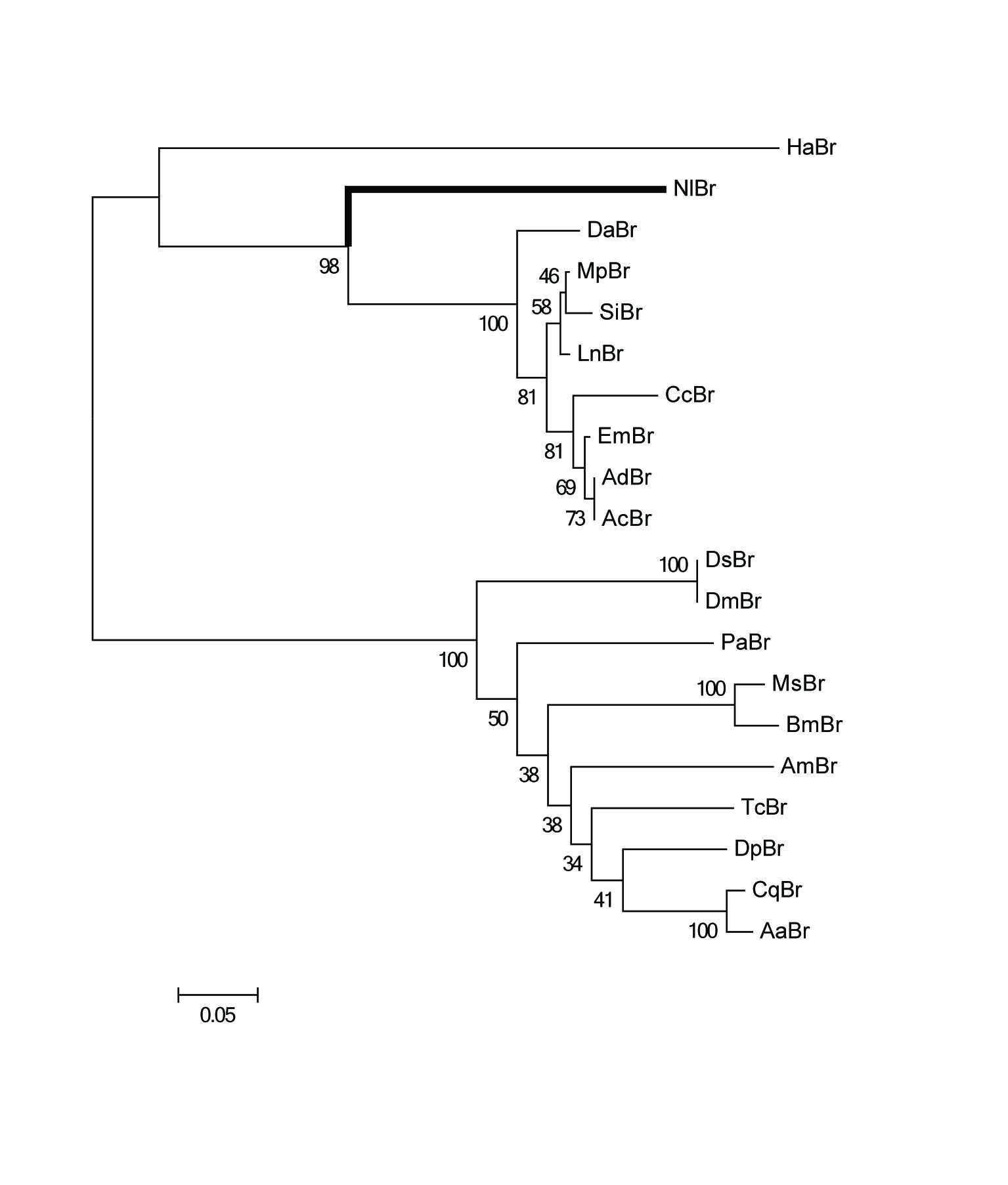

Supplement: Figure S1 — Phylogenic analysis of Br proteins. Ln, Lasius niger (KMR04998.1); Em, Eufriesea Mexicana (XP_017755324.1); Ac, Apis cerana (XP_016913178.1); Si, Solenopsis invicta (XP_011169636.1); Mp, Monomorium pharaonic (XP_012538438.1); Da, Diachasma alloeum (XP_015108610.1); Ad, Apis dorsata (XP_006623320.1); Pa, Pyrrhocoris apterus (AEW22982.1); Ha, Helicoverpa armigera (AGW25457.1); Ds, Drosophila simulans (KMZ07685.1); Dp, Diploptera punctate (AIM47237.1); Cq, Culex quinquefasciatus (EDS39011.1); Am, Apis mellifera (NP_001035356.1); Aa, Aedes aegypti (AAS80329.1); Cc, Ceratina calcarata (XM_018020932); Bm, Bombyx mori (NP_001104804.1); Tc, Tribolium castaneum (BAJ16548.1); Dm, Drosophila melanogaster (ACZ95175.2); Ms, Manduca sexta (AAC78288.1); Nl, Nilaparvata lugens (KU640204). [file Image1.JPEG]

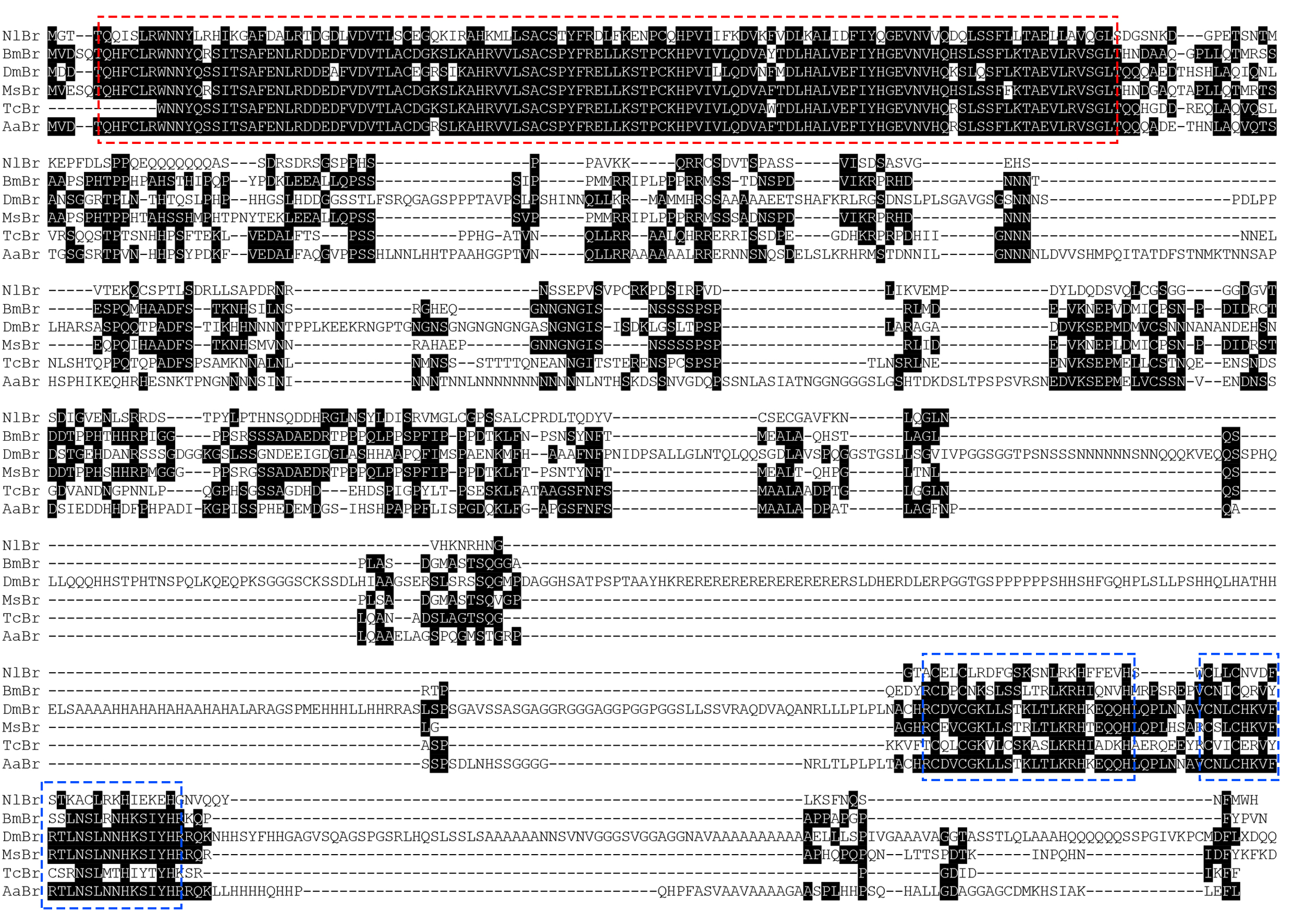

Supplement: Figure S2 — Alignment of the amino acid sequences of N. lugens Br with those of its homologs in Bombyx mori, Drosophila melanogaster, Tribolium castaneum, Manduca sexta and Aedes aegypti. Domains-BTB (Red), C2H2 Zn finger (Blue) are boxed. Bm, Bombyx mori (NP_001104804.1); Dm, Drosophila melanogaster (ACZ95175.2); Tc, Tribolium castaneum (BAJ16548.1); Ms, Manduca sexta (AAC78288.1); Aa, Aedes aegypti (AAS80329.1). [file Image2.JPEG]
